# The role of diet in cancer: the potential of shaping public policy and clinical outcomes in the UK

**DOI:** 10.1186/s12263-024-00750-9

**Published:** 2024-08-03

**Authors:** Oliver Britten, Sabrina Tosi

**Affiliations:** 1 Barts and The London School of Medicine and Dentistry, Queen Mary University of London, Turner St, London, E1 2AD UK; 2 Leukaemia and Chromosome Laboratory, College of Health, Medicine and Life Sciences, Brunel University London, Kingston Lane, Uxbridge, UB8 3PH UK

**Keywords:** Diet, Cancer, Prevention, Treatment, Dieticians, Public policy, Personalised nutrition

## Abstract

Cancer universally represents one of the largest public health concerns, substantially contributing to global disease burden and mortality. The multifaceted interplay of environmental and genetic factors in the disease aetiology and progression has required comprehensive research to elucidate modifiable elements which can reduce the risk of incidence and improve prognosis. Among these factors, diet and nutrition have emerged as the most fundamental with a significant potential for influence and effect. Nutrition is not only an essential part of human survival, but also a vital determinant of overall health. Certain dietary requirements are necessary to support normal physiology. This includes individualised levels of macronutrients (proteins, carbohydrates and fats) and specific micronutrients (vitamins and minerals). Extensive research has demonstrated that diet plays a role in cancer pathogenesis at the genetic, epigenetic and cellular level. Therefore, its potential as a modifiable determinant of cancer pathogenesis for the purpose of prevention and improving management of disease must be further explored and implemented. The ability to influence cancer incidence and outcomes through dietary changes is underutilised in clinical practice and insufficiently recognised among the general public, healthcare professionals and policy-makers. Dietary changes offer the opportunity for autonomy and control over individuals health outcomes. Research has revealed that particular dietary components, as well as cultural behaviours and epidemiological patterns may act as causative or protective factors in cancer development. This review aims to comprehensively synthesise this research to further explore how to best utilise this knowledge within the community and clinical environment for more effective cancer prevention and therapeutic strategies. The identified key areas for improvement include the development of more specific, widely accepted guidelines, promoting increased involvement of dieticians within cancer multidisciplinary teams, enhancing nutritional education for healthcare professionals and exploring the potential implementation of personalised nutrition tools. A greater understanding of the complex interactions between diet and cancer will facilitate informed clinical interventions and public health policies to reduce global cancer burden and improve care for cancer patients and survivors.

## Background

Cancer remains a leading cause of mortality and morbidity worldwide and in the UK. With many projections indicating a continued increase in incidence, experts are advocating for preventative measures to be put into effect to tackle the greatest risk factors [1–4].

Over 37% of cancer cases in the UK are attributable to modifiable risk factors, with diet being the second-highest contributor after tobacco. Additionally, some studies have shown as high as 35% of all cancer-related mortalities are associated with diet [5–7]. The identification of numerous potentially carcinogenic and chemoprotective agents, has initiated a multitude of strategies aimed for chemoprevention through the inclusion and elimination of dietary components and nutrient supplementation. While there is some promising evidence suggesting the slowing, prevention and reversal of carcinogenesis, further research is required for implementation into clinical practice and public policy [8–10]. Nutritional and dietary recommendations for cancer prevention and cancer patients, including from the Eatwell guide, the World Cancer Research Fund (WCRF) and the European Society for Clinical Nutrition and Metabolism (ESPEN), predominantly remain aimed at maintaining a healthy weight, meeting nutritional requirements, increasing consumption of vegetables and limiting intake of alcohol and processed foods [11–15]. This guidance provides a crucial framework for a healthy diet, with the intention of preventing and controlling non-communicable diseases including cancer. However, it is essential to strongly consider inclusion of more specific, consensus-driven recommendations for subgroups such as those identified as high-risk of developing cancer, individuals with cancer or cancer survivors. Approximately half of all cancer patients modify their diet following diagnosis with the aim of improving therapeutic outcome and preventing recurrence [16–20]. Some of the more popular diets chosen by cancer patients include vegan, paleolithic, alkaline, macrobiotic and ketogenic [16]. Without proper guidance and the use of evidence-based research, patients risk making nutritional decisions, including extreme changes in diet or supplementation, which may interact with their cancer treatment or cause additional health issues as a result of malnutrition. Therefore, there is a requirement for clearer guidance for these individuals.

## Cancer metabolism

To comprehend the significance of the role of nutrition in cancer incidence and prognosis, it is crucial to establish an understanding of cancer metabolism. Cancer has a range of metabolic demands which are influenced by genetic and epigenetic factors, the tumour microenvironment, the type of tissue which underwent malignant transformation and the individual’s metabolism. Nearly 100 years ago, Otto Warburg made the observation that cancer tissue utilises higher levels of glucose and produced more lactate than non-malignant tissue, even in oxygenated environments. This phenomenon of aerobic glycolysis was termed the Warburg effect [21, 22]. There are many proposed mechanisms as to why the Warburg effect is advantageous for tumorigenesis despite the decreased production of ATP in comparison to oxidative phosphorylation. One potential explanation is that it serves as an evolutionary mechanism to combat the hypoxic conditions caused by the blood supply lagging behind the high rate of growth of the tumour. It was also proposed that the by-products of glycolysis could be utilised for the support of cancer growth through metabolite synthesis, including amino acids, nucleotides and lipids. Studies have demonstrated these metabolites also function as signalling molecules to promote tumorigenesis through the regulation of gene expression [23]. Additionally, the production of reactive oxygen species as a result of aerobic metabolism has been shown to be beneficial for cancer growth by inducing DNA damage [24]. The growing field of literature and recent findings have indicated the significance of nutritional supply in cancer development and treatment outcomes. Further research into the metabolic changes cancer cells undergo may expose mechanisms which can be exploited for therapeutic purposes or cancer prevention. For instance, specific dietary modifications may hold the potential to prevent or inhibit cancer development or growth. Furthermore, it is important to investigate how these nutritional changes can be harnessed to optimise the efficacy of the currently utilised chemotherapies.

## Nutrigenomics

Nutrigenomics investigates the influence of nutrition on gene expression and chromatin structure, consequently exploring the mechanisms by which specific dietary patterns affect cellular metabolism and the expression of proteins and other metabolites. One of the aims of nutrigenomics is to identify biomarkers that undergo alterations in response to dietary factors, thereby facilitating the early detection of disease and high-risk individuals. The field of nutrigenomics is highly conducive for understanding the relationship between diet and cancer risk on an individualised level [25]. As a result of recent advancements in genome sequencing, individuals with specific single nucleotide polymorphisms (SNPs) have been shown to have altered cancer risk depending on certain dietary changes [26]. For instance, carriers of a homozygous variant of the C677T polymorphism of the *MTHFR* gene exhibited decreased risk of colorectal cancer when their diets consisted of low levels of folate and alcohol consumption. On the other hand, some studies have shown individuals with a specific SNP of the *NAT* genes, which encodes the N-acetyltransferase enzymes, are at higher risk of bowel cancer when increased amounts of red meat are consumed, thus intensifying the carcinogenic impact of this dietary component [26, 27]. While there are a growing number of findings displaying an interaction between dietary factors and genetics in cancer risk, there has been inconsistencies and difficulty in reproducing many of these findings. This is potentially a result of variable levels of exposure to the particular dietary constituents, interactions with other components of the diet or other genetic or physiological variation affecting digestion or absorption. Variability of genomes, epigenetics, proteomics and microbiomes within the population can influence the physiological response to bioactive compounds in food [28]. Considering that SNPs constitute 90% of the variability in human genetics, further understanding of these polymorphisms may prove vital in elucidating this variation in response to nutrition. These genetic differences result in phenotypic variation, affecting the physiological response to food components, including tolerance, metabolism and even taste preference. For example, the *SLC2a2* gene encodes the glucose transporter type 2 which is involved in glucose regulation. Individuals harbouring a SNP in the *SLC2a2* gene exhibit elevated levels of sugar consumption [29] which may encourage cancer growth and progression [30]. Although nutrigenomics is a promising field to identify those who would derive the greatest benefit from specific dietary changes and for offering personalised dietary recommendations to mitigate cancer risk, further research and advancements are required to be able to utilise these findings for the application of personalised diets for cancer prevention.

## Epigenetics

Epigenetic research has indicated that exposure to environmental factors pre-conception, during gestation and throughout our lifespan, influences risk of cancer development [31]. Cancer develops as a result of accumulation of genetic and epigenetic modifications [32]. A growing body of evidence has demonstrated that the bioactive constituents in our diet plays a role in altering the epigenome. A significant portion of these components display protective properties and have the potential to contribute to cancer prevention. Additionally, recent evidence indicates that some dietary elements have the capacity to modify the epigenome to reverse abnormal gene activation or suppression [33]. The introduction of these dietary components, and the exclusion of procarcinogenic compounds, may be implemented into an individual’s diet for cancer prevention or to complement therapeutic approaches. Dietary constituents containing phytochemicals, including cruciferous vegetables, garlic, and some fruits and herbs, are widely recognised for their role in protecting against cancer development [34, 35]. It is now acknowledged these phytochemicals act as epigenetic modulators which negatively influence tumour development and progression [36].

Epigenetic alterations typically arise as changes in DNA methylation, non-coding RNA, RNA interference and histone modifications [33]. Detection and monitoring of these changes can serve as a powerful clinical tool, offering a novel method to facilitate early diagnosis and for prognostic evaluation [37]. The most promising aspect of exploring epigenetics for cancer prevention and outcome, is that unlike genetic mutations, epigenetic modifications are reversible. Hence, through incorporation of dietary changes, there exists the opportunity to reverse epigenetic changes which were previously causing oncogene activation or tumour suppressor gene silencing. Research has demonstrated DNA methylation is involved in the regulation of gene expression, primarily through silencing gene transcription. Aberrant DNA methylation can include hypermethylation and hypomethylation, both of which have been identified in virtually all cancer types. DNA hypermethylation has been linked with the inactivation of a growing number of powerful tumour suppressor genes, such as *p16INK4a*,* MLH1*,* RB1* and more than 600 others [37, 38]. Additionally, genome-wide hypomethylation is a characteristic epigenetic finding in cancer. It is thought to cause chromosomal instability and aberrant gene expression. Hypomethylation has also been linked with upregulation of oncogenes in cancer cells, including *MYC* and *BCL-2* in chronic lymphocytic leukaemia [39, 40]. Some studies have shown promising results indicating the potential implementation of hypomethylation marker detection to aid with cancer surveillance, diagnosis and treatment [41, 42]. Recent evidence indicates that DNA methylation influences cancer cell metabolism and that diet can change the level of methylation in tumours [43]. DNA methylation necessitates the intake of micronutrients referred to as methyl donors, these include methionine, choline and folate. Therefore, there is potentially a direct effect on the degree of methylation and consequently, on the development of cancer, by modifying the levels of these nutrients consumed. This has been shown in a mouse-model whereby a methyl-donor deficient diet was protective against bowel cancer [44].

In addition to abnormal DNA methylation, aberrant histone modifications and dysregulated chromatin structure has also been implicated in tumorigenesis [37]. The patterns of histone modifications are responsive to environmental factors, including diet, which can alter gene expression and DNA repair mechanisms. Hence, dietary elements may modify chromatin remodelling and the profiles of histone modifications, hindering DNA repair mechanisms [37].

## Epidemiology and food anthropology

While there is an increasing cancer burden globally, variation in patterns and prevalence of cancer types have been observed across the world [4]. This observation suggests the presence of significant environmental factors contributing to the development of cancer which varies among different populations. Many of these epidemiological studies revealed a strong correlation between the prevalence of certain cancer types and dietary factors. For example, countries with greater consumption of red meat have been associated with increased rates of colorectal cancer [45]. To add to this complexity, these patterns of cancer evolve over time, influenced by many factors, including migration and education [46]. One of the challenges of this research is the difficulty of analysing diet as an isolated environmental factor. This is often not possible and is why these epidemiological studies are valuable for identifying these potential dietary factors.

In addition to the effect of the dietary constituents on cancer risk, the method of cooking and storage of food must be considered. The practice of utilising salt for food preservation is frequently used in southern China, South East Asia, Japan and certain regions of northern Africa and the Middle East. Consumption of these salt-preserved foods has been associated with increased risk of nasopharyngeal and stomach cancer. As a result, the prevalence of stomach and nasopharyngeal cancer is notably higher in these regions [9, 47, 48]. The rationale behind this elevated cancer risk has been theorised to be caused by the increased salt intake, the presence of nitrates in these salt-preserved foods, or the heightened susceptibility to Helicobacter pylori infection, which is a known risk factor for stomach and oesophageal cancer [9]. Studies have demonstrated that in some of these regions, such as China, increasing the consumption of fruits and vegetables, primarily to elevate vitamin C levels, has led to reduced rates of stomach and oesophageal cancers [49]. Vitamin C has been the subject of ongoing research for cancer prevention and treatment, yielding results of variable efficacy. Recent discoveries have suggested a role in immunomodulation via newly unveiled signalling pathways as well as established epigenetic and antioxidant effects [50, 51]. Current research suggests that while pharmacological concentrations of intravenous vitamin C may have standalone antitumour qualities, its potential benefits could be enhanced when utilised in conjunction with immunotherapies, such as CAR-T therapy, PD-1 inhibitors and other checkpoint inhibitors [50].

Nearly half a century ago, epidemiological studies suggested that the reduced prevalence of colorectal cancer in specific regions in Africa were attributable to elevated dietary fibre intake [52]. Recent findings show impressive reductions in the occurrence and mortality rates of colorectal, oesophageal and breast cancer when the daily fibre intake exceeds 25–29 g [53]. Intriguingly, the source of this fibre appears to affect the efficacy of its anti-cancer properties with some studies showing fibre from whole grain foods being slightly more effective than from fruits and vegetables [53]. However, these encouraging findings may be enhanced by a combination of other factors, such as variable glycaemic load, BMI, age, total carbohydrate intake and other dietary factors. Fruits and vegetables contain additional nutrients besides fibre which are cancer protective [54]. As little evidence is currently available, sources with naturally occurring dietary fibre such as fruits, vegetables and whole grains should be chosen preferentially over more highly processed sources such as those found in many breakfast cereals. Dietary fibre is predominantly metabolised by the local microbiota in the large intestine in a process called fermentation. These native microorganisms and their genetic material, the microbiome, within the gastrointestinal tract are involved in many vital processes relating to the immune system, metabolism and synthesis of important nutrients as well as in the central nervous system via the gut-brain axis. This fermentation of dietary fibres by the microbiota has also been shown to have anticancer properties [55–57].

## Microbiota and microbiome

Recent data suggest that alterations in gut microbiota and microbiome induced by dietary changes play a significant role in regulating various processes involved in cancer development, progression and response to chemotherapy and immunotherapy [58–61]. Therefore, these interactions between diet and microbiome may be utilised for cancer prevention and to improve outcomes alongside cancer therapy. Although gut microbiota typically remains relatively consistent, numerous factors can influence the composition, diversity and quantity of specific microorganisms. Among these factors, diet is the most easily modifiable with significant potential for facilitating improved outcomes and cancer prevention [62]. It is established that diet has a substantial influence on the composition of the gut microbiota, with dietary fibre and phytochemicals playing an important role. There are many positive effects of fruits, vegetables, whole grains, spices and teas, including supporting growth and maintaining balanced populations of microbiota.

It is believed that the microbiome is more readily adaptable and influenced by external factors, including diet, in comparison to innate properties derived from the host. This rapid adaptability provides the potential to manipulate how the microbiome participates in and facilitates metabolism, absorption and even nutrient synthesis [63]. Hence, diet influenced changes in gut microbiome may be utilised to alter individuals physiology and consequently, impact cancer pathogenesis and progression. It has been established that individual specific variation in gastrointestinal microbiota can affect the physiological response to dietary changes [64]. Efforts are being made to identify markers within individuals microbiome to enable personalised nutritional strategies to facilitate the most beneficial responses to dietary alterations [65–67]. However, the complex interplay between variations in human physiology, microbiome and the influence of environmental factors such as diet, make creating a personalised tool challenging. In the future, this approach could serve as an additional resource to maximise the benefits of dietary changes for the purpose of cancer prevention and enhancing treatment outcomes.

One of the mechanisms of microbiome involvement in tumour etiology and progression is the synthesis of chemoprotective and carcinogenic compounds. Currently only several of the tens of thousands of the metabolites have been studied for their involvement in cancer development. However, there are likely many more molecules to play a role in tumorigenesis that are yet to be identified [61, 68]. One of the anti-cancer microbiome bio-products are short chain fatty acids (SCFAs), produced by dietary fibre fermentation. While fibre plays an important role in cancer prevention irrespectively, SCFAs, notably butyrate, are involved in glucose metabolism, immunomodulation and other homeostatic mechanisms essential for maintaining health [69]. Studies have demonstrated that SCFAs can inhibit cancer growth across several cancer types through modulation of the cell cycle as well as other signalling and metabolic pathways. Additionally, when combined with chemotherapeutic medications, SCFAs have displayed enhanced treatment efficacy in colorectal cancer [70–72]. Dysbiosis and overgrowth of certain microbial species has been linked with elevated risk of cancer development. Consumption of fat significantly affects the composition of the gut microbiome [73]. Studies in mice have shown that diets with high levels of fat result in an increase in the quantity of specific species which have been shown to reduce T cell response to cancer cells, thus reducing the immune response against tumour development. Elevated fat intake also leads to increased bile acid secretion which is modified by the microbiome to form carcinogenic metabolites [61, 74]. Research into the involvement of dysbiosis in chronic inflammation and cancer have identified specific microbial species which exhibit anti-cancer and anti-inflammatory properties. These are predominately found in individuals with a healthy bodyweight who consume diets with sufficient fibre and foods with a low glycaemic index. Therefore, diets such as the Mediterranean diet, which promote this nutritional intake, could be utilised to reduce cancer incidence by altering the composition of the microbiome. Prebiotics, probiotics, and combined formulations, known as synbiotics, have also been implemented in an attempt to promote growth of anti-inflammatory and anti-cancer bacterial species for cancer prevention and treatment. Research to date has yielded variable and inconsistent results [75–78]. However, here have been some promising preclinical studies in utilising microbiota changes for oncosuppression and as an adjuvant therapy. In mouse models, prebiotics were used to prevent growth of melanoma [79]. Additionally, consumption of synbiotics reduced proliferation of cells within the colon in patients with colon cancer as well as improved the function of the colonic epithelium and reduced DNA damage in intestinal cells in individuals who have had polyps removed [80]. However, additional research, particularly in humans, is required to validate the efficacy of microbiome altering substrates for cancer prevention and treatment.

**Fig. 1:**
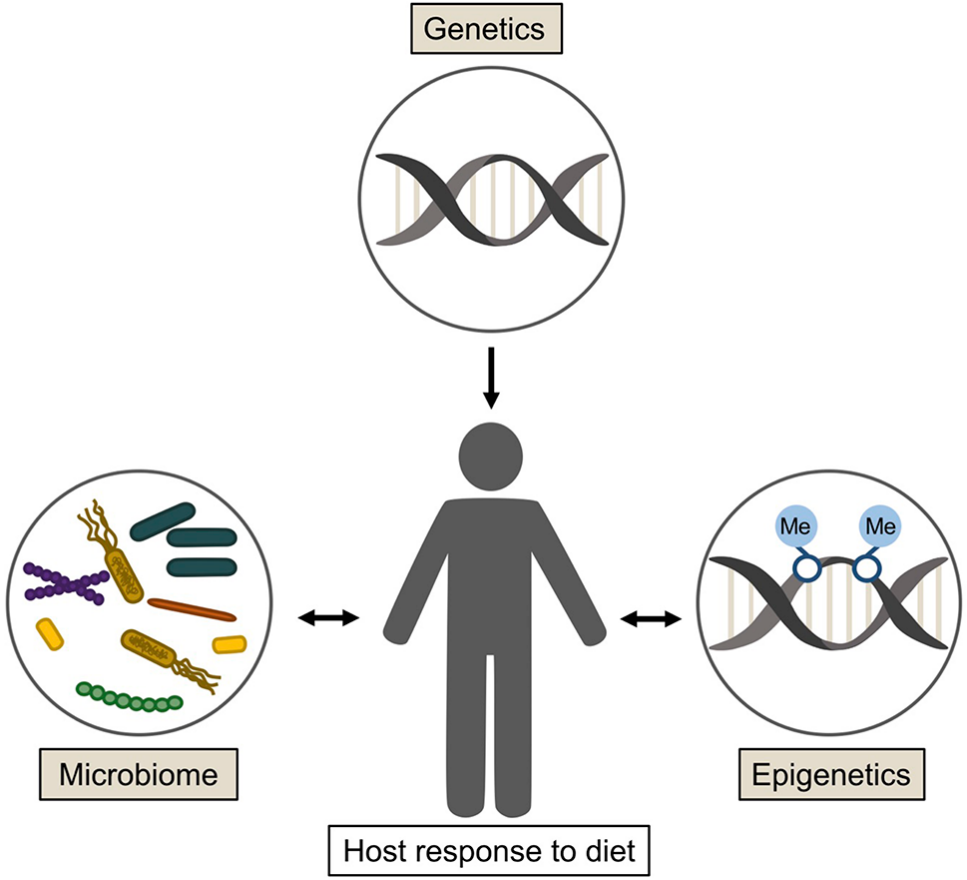
An overview of the factors influencing individual response to dietary components. Response to diet varies between individuals based on a number of factors including the hosts genetics, epigenetics and microbiome. While these factors influence host response to nutritional changes, dietary changes also have the capacity to alter composition of the microbiome and epigenetics

Microbiome alterations resulting from dietary adjustments have been demonstrated to influence drug metabolism and response to therapeutic interventions. Dietary factors affecting pharmacokinetics have many well-established mechanisms, including inhibition and activation of cytochrome P450 enzymes [81, 82]. Currently, there is little research into these dietary and microbiome effects on chemotherapeutic interventions. However, research has shown that androgen deprivation in mouse models result in an altered microbiome composition and an increase in androgen metabolism by this modified microbiota which consequently increased prostate cancer growth and resistance to androgen-deprivation therapy which is a mainstay of prostate cancer treatment [83]. Therefore, microbiome manipulation may provide improved outcomes alongside chemotherapeutic strategies. Additional investigation is necessary to elucidate the mechanisms of these alterations in drug metabolism and response as well as the impacts of these therapies on the microbiome. A summary of the factors influencing host response to diet is shown in Fig. 1.

## Challenges and recommendations

At this time, uncertainty remains as to which dietary approach is most efficacious for cancer prevention, to improve therapeutic response or as an adjuvant therapy. Furthermore, the concept of developing a novel conclusive diet for this purpose appears unviable. Based on the available research, it is clear that the effectiveness of a diet varies depending on individual physiology, genetics, epigenetics, the microbiome, as well as cancer type and therapeutic intervention. In order for any intervention to be effective, individuals need to be capable of adhering to it. Therefore, personalisation is crucial for dietary guidance to be successful. Irrespective of efficacy, it is essential to consider cultural habits, religious beliefs, personal values, preferences and allergies. This calls for adaptability, sufficient time with trained healthcare professionals (HCPs), and a willingness to embrace lifestyle changes [10].

### Future guidelines

The breath of this topic presents considerable challenges in summarising, categorising and evaluating the clinical relevance of the current research. Determining the value of integrating these findings into guidance, public policy or clinical practice adds further complexity [84]. These studies vary widely in dietary intervention implemented, population, timeframe, cancer type, additional therapies, tested outcomes and how these outcomes are determined. Moreover, heterogeneity is observed in research investigating intervention with similar or identical dietary components. Therefore, employing more consistent and standardised criteria, with well-defined variables and evaluation methods for outcomes, will help improve the reproducibility of these results and their integration into clinical practice and wider public policy [84]. One of the challenges of this is that in contrast to nutritional studies for conditions like diabetes and cardiovascular disease which have well-defined and recognised markers of disease, such as blood glucose and cholesterol level respectively, cancer has fewer definitive markers to measure outcomes [26]. The lack of sufficiently compelling findings has led expert groups such as ESPEN, the WCRF and the International Agency for Research on Cancer, to concentrate on broad recommendations [9, 85]. Future research will hopefully elucidate new, and already identified dietary components which have insufficient evidence, as protective or causative factors for inclusion in future guidelines. The European Institute of Oncology’s ‘SmartFood’ program, is a project aiming to bridge this disparity between research and clinical practice. With a focus on investigating dietary modulation for cancer prevention, this initiative combines the knowledge and experience of researchers, nutritionists and clinicians for the shared goal of elucidating the connection between diet and health and increasing awareness of effective nutritional intervention [86]. The implementation of similar programs within the UK could prove to be beneficial. More specific nutritional guidelines are imperative for cancer prevention, cancer patients and cancer survivors, as these separate groups have varying requirements, priorities and challenges.

An evaluation was conducted to assess the standard and homogeneity of European dietary guidelines for cancer survivors [85]. The study revealed that the guidelines consistently recommend diets rich in fruits, vegetables and fibre, while advising against excessive consumption of red and processed meat. These recommendations are analogous to the nutritional guidelines for cancer prevention [87]. This is in contrast to the Western dietary pattern, characterised by high intake of sugar, high-fat dairy and processed and red meat, which has been associated with increased risk of breast, prostate, gastric, colorectal, pancreatic and lung cancer when compared to a Mediterranean or prudent diet [88–95]. Although the correlation between the Western diet and cancer is multifactorial, the association between this dietary pattern and obesity should not be overlooked. Weight management was also a consistent topic within guidance, as excess body fat is associated with higher prevalence and poorer outcomes in several cancer types. In fact, obesity is responsible for causing around 4–8% of all cancer cases [96]. Therefore, maintaining a healthy bodyweight, as defined by the World Health Organisation as a body mass index (BMI) of 18.5 to 24.9 [97], decreases the risk of developing at least 13 different cancer types, including breast, colorectal, stomach and liver [96]. There is conflicting evidence on the effect of excess body weight on outcomes in cancer patients. With some studies suggesting an elevated BMI may be associated with more favourable prognosis in some cancers [98], while others claim the opposite [99]. A higher BMI may affect prognosis indirectly by increasing surgical risk or resulting in suboptimal therapeutic dosages [100].

### Nutritional training for healthcare professionals

While there were several recurring recommendations, the clinical applicability of many current guidelines was deemed to be low [85]. This indicates that many of these lacked the information necessary for HCPs to integrate this advice into clinical practice. In order for guidelines to be effectively integrated, sufficient direction on their application in clinical practice is key. According to Kleaver et al., of the guidelines investigated, the highest scores in this area were achieved by ESPEN and WCRF [85, 101, 102]. Some of the main obstacles for integration of these guidelines appears to be insufficient training on nutrition and, consequently, many clinicians lack the required knowledge. Additionally, inadequate consultation times pose a barrier, resulting in limited time to engage in dietary discussions with patients [103]. A potential strategy for combatting this is increased nutritional teaching at medical school which can be further built on throughout training pathways. According to a study conducted with UK medical students and doctors, almost all participants agreed that doctors have a responsibility to provide nutritional care and recognise that diet is an important factor for both health and development of disease. Less than half of respondents indicated receiving nutritional training in the past year. Among these, more than 70% reported receiving less than 2 hours of teaching on nutrition. As a result, only 26% of doctors felt confident in their knowledge. Consequently, the majority of them provided diet-related information less than once a month [104]. A study investigating nutritional guidance provided by HCPs for cancer patients within the UK demonstrated that while some discussion and information about diet may be provided by different HCPs, there was a significant lack of familiarity with the appropriate guidance [105]. Fortunately, most participants expressed they would welcome additional teaching on diet and nutrition. Therefore, there has been an expressed need and willingness for further inclusion of nutrition within the curriculum. Although the General Medical Council lists the ability to “discuss the role and impact of nutrition to the health of individuals and society” as a requisite skill of qualified doctors within the UK, this outcome is evidently not currently satisfied by the majority of clinicians [106].

### Dieticians: a member of the multidisciplinary team

Nutritional guidance is an essential part of providing patient-centred care. Despite the growing understanding of the role of diet in cancer prevention and care, awareness remains limited among patients, HCPs, administrators and policy-makers. The European Health Union developed the ‘Europe’s Beating Cancer Plan’ which primarily aims to further implement a shift towards an integrated, holistic, patient-centred approach to cancer prevention and care. Nutrition is a key part of this plan and was recognised as an evidence-based, cost-effective approach which provides a significant improvement on health outcomes and quality of life. One of the predominant findings was the lack of incorporation of expert provided, evidence-based nutritional guidance within the multidisciplinary team (MDT). This insufficient integration of dieticians within the MDT was identified within Europe, and across the globe [107, 108]. The same is to be said with the National Health Service (NHS) and the UK. Research has already highlighted the need for nutritional guidance and for the incorporation of dieticians into oncology clinics and cancer treatment within the UK [109]. Advocacy has been expressed for altering national guidelines for the integration of specialist dieticians into oncology MDTs, including for head and neck cancer. Further recommendations included the implementation of a validated nutritional screening tool to identify patients at high-risk of malnutrition, and providing early referral to dieticians accordingly [110, 111]. Encouraging early and prompt nutritional support alongside regular nutritional evaluations are additional recommendations which can be implemented into all oncology MDTs. In addition to their role in improving quality of life, therapeutic efficacy and prevention of relapse, a dietician can help tackle many challenging adverse effects of chemotherapeutic agents. Almost all anti-cancer therapies have unwanted side-effects, including nausea, vomiting, diarrhoea, reduced appetite and taste alterations, which can negatively impact the patients’ relationship with food and intake. Patients may even continue to be affected by these adverse effects after the completion of treatment, resulting is both physiological and psychological impact [103, 112]. This further indicates the need for a multidisciplinary approach which incorporates experts in oncology and nutrition.

**Fig. 2:**
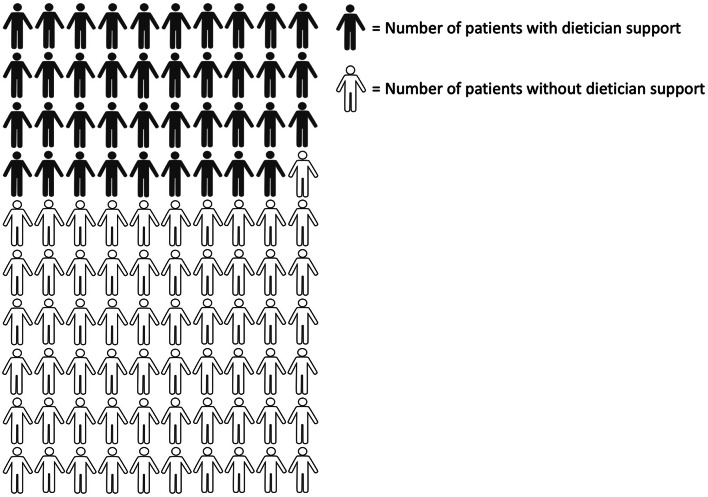
A small percentage of cancer survivors receive nutritional guidance from a dietician in the UK. According to Sullivan et al. [18], only 39% of cancer survivors ever received nutritional guidance from a registered dietician at any stage from diagnosis onward

Although 89% of cancer survivors in the UK consider nutrition as a ‘very/extremely’ important component of their treatment and care, fewer than 40% reported having seen a dietician (Fig. 2). Among those who received input from a dietician, 93% found the guidance beneficial. This perceived benefit was found to be similar across the eight cancer types surveyed. The predominant reasons for not being assessed by a dietician included not receiving a referral and a lack of awareness of the available service. The majority of those who didn’t receive guidance from a dietician conveyed a desire for more nutritional support [18]. These findings further demonstrate the perceived advantage of a dietician’s support and the need to address these unfulfilled requirements of patient-centred care.

The implementation of dietary changes alongside and post-treatment appears to be an effective time for coherence with recommendations [113, 114]. Individuals have been found to be more receptive and more likely to acknowledge the importance of appropriate nutrition following a cancer diagnosis [105, 113, 115]. This increased motivation and readiness to adopt these dietary recommendations should be supported and utilised. Equally, this increased susceptibility and desire to receive nutritional information creates an elevated risk of worry and confusion caused by the vast amount of unreliable sources. Therefore, HCPs are the preferred source of these recommendations, a preference shared by cancer survivors [116]. However, as a result of insufficient incorporation of dieticians within the MDT and limited resources within the UK, very few cancer patients and survivors are referred to the support they require [18, 117]. The growing number of cancer cases and survivors in England has created additional pressure on this system. Access to dietary guidance and care is an integral component of the NHS England National Cancer Strategy [118]. However, the shockingly low reported numbers of patients receiving this care highlights the need for further changes to be implemented and resources to be added.

### Personalised nutrition

**Fig. 3:**
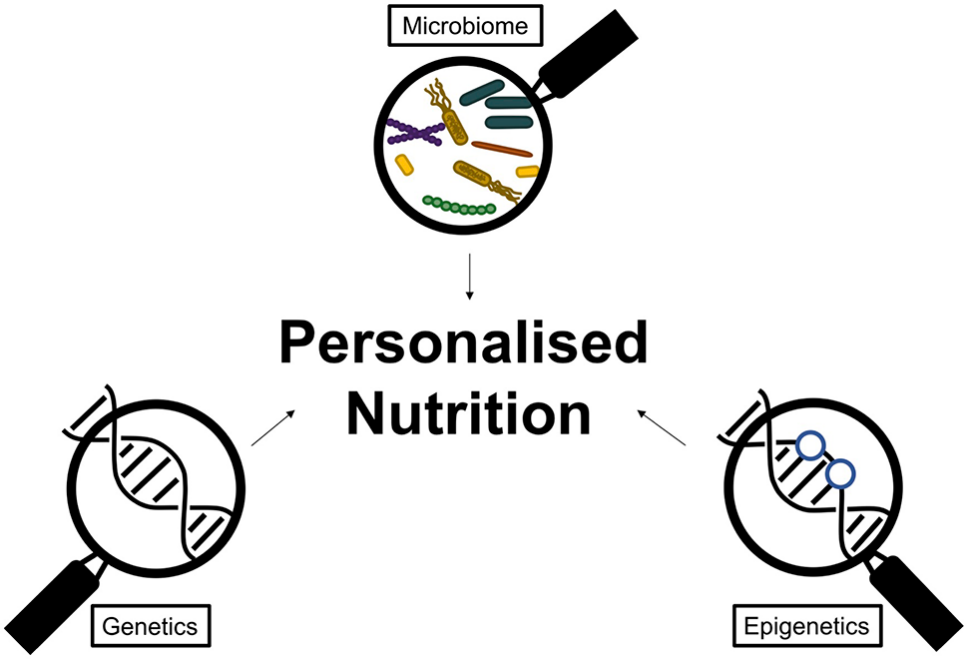
Overview of promising parameters to be used in personalised nutrition testing. These include tools investigating an individual’s microbiome composition, genetics and epigenetics to offer tailored nutritional guidance for optimal response to dietary changes

The existing nutrition guidelines target the broader population. While this enables accessibility to a wider audience, it does not facilitate modifications to accommodate for personal preferences, culturally appropriate choices, allergies, and individual physiological responses to dietary components. However, recent advances now make it possible to offer personalised and optimised guidance through the identification of high-risk subpopulations and tailoring individual recommendations. These recent advancements include tools which enable the identification of genetic and epigenetic markers which indicate specific nutritional susceptibilities [119, 120]. Microbiome analysis to predict individual responses to dietary components are also available [121]. The combination of genetic, epigenetic and microbiome testing could potentially facilitate personalised diet plans to optimise health outcomes. Currently, this is only commercially available in the UK and is not offered by the NHS. Implementation of these tools in the future should be strongly considered and may be highly beneficial. The involvement of HCPs in this service would likely facilitate the effective utilisation and incorporation of this information into individual lifestyles. Although these personalised nutrition strategies have been demonstrated to cause behavioural shifts and improve outcomes [122, 123], the efficacy of these personalised nutrition strategies have been challenged, primarily due to lack of compelling evidence. The research available investigating the efficacy of some of these tools, primarily the nutrigenomic focused personalised diets, have yielded inconsistent results [124]. The variability of these findings is anticipated due the presence of a multitude of factors influencing health outcomes asides from genetics, including age, epigenetics, microbiome as well as other behavioural and lifestyle components. Consequently, a comprehensive personalised nutrition approach that incorporates multiple of these factors could improve the effectiveness and consistency of this strategy,see (Fig. 3).

Artificial intelligence (AI) is rapidly becoming increasingly integrated into healthcare. Its application in personalised nutrition has the potential to transform the integration of these various factors and growing insights, streamlining the process for patients and clinicians, all while optimising health outcomes. Utilisation of AI to analyse extensive datasets could facilitate the identification of nutritional requirements through analysis of individuals’ genetics, microbiome and epigenetics. This may also uncover novel connections between diet and cancer. Personal AI-enabled devices may hold the potential to assist diet monitoring, provide tailored feedback and enhance the tracking experience for individuals [125], further improving behaviour change and coherence. AI techniques can be used in dietary assessment and analysis through automated detection of food products from pictures and dietary records [126]. This approach could simplify and facilitate dietary assessments through the implementation of deep learning models for image analysis to help understand and improve eating habits. However, these methods still require further research and validated databases to improve accuracy [127]. Approaches such as these hold the potential to transform clinical nutrition through leveraging AI and digital tools for real-time diet monitoring. They could also be utilised for personalised interventions, cancer prognostics and identification of high-risk groups through predictive models. These models apply machine learning to detect patterns within large datasets, including information on diet, lifestyle factors, genetics, and epigenetics, which can indicate the probability of the development of disease and prospective prognosis [128, 129]. These tools may be used to facilitate nutritional screenings for the identification of cancer patients who are malnourished or at high-risk of malnutrition, as well as predicting outcomes in intensive care [130–132]. Additionally, these methods may elucidate the relationship between obesity and other co-morbidities such as diabetes and hypertension with cancer, helping shape and prioritise interventions [129, 133]. An AI-driven nutritional assistant, to function as a virtual dietician for cancer patients, has been developed to increase access to guidelines-based nutritional support. This provides a potential solution to address the lack of access to dieticians as a result of an insufficient workforce [134]. The implementation of digital health tools continues to grow in prevalence within the UK, with digital transformation named as a ‘top priority’ for the Department of Health and Social Care and the NHS [135]. A randomised control trial was performed to assess the efficacy of varying levels of digitally delivered personalised nutritional guidance against a more conventional “one size fits all” approach to improve dietary habits. This study, conducted across multiple European countries, along with similar research, revealed that personalised dietary interventions resulted in a higher rate of positive dietary changes when compared to non-personalised advise [136, 137], thus demonstrating the effectiveness of digital health tools within this space.

Integration of AI into healthcare is complex and rapidly evolving, necessitating careful consideration of ethical and legal implications. AI-based devices or applications designed for treating or preventing conditions like cancer are classified as ‘software as a medical device’ [125, 138]. Ensuring effective governance and regulation is crucial for the utilisation of AI in personalised nutrition, from both a data management and a clinical implementation perspective. Moreover, if the datasets used to train AI models are inadequate or biased, the system may continue to reinforce existing inequalities within healthcare. Therefore, it is essential to utilise diverse databases that encompass information from different genders, socio-economic, cultural and ethnic demographics [125].

Future direction of research is primarily focused on enhancing personalised nutrition for the purpose of improving overall health, preventing and combatting diseases such as cancer. In the United States, the National Institute of Health has begun a 10 years personalised nutrition research initiative running through to 2030. This programme aims to integrate research on genetics, microbiome, and other factors that impact how individuals respond to diet and their susceptibility to conditions like cancer. The goal of this strategy is to leverage these findings, along with AI research and clinical training, to advance personalised or ‘precision’ nutrition [139]. The UK equivalent agency, the National Institute of Health and Care Research, initiated the Cancer and Nutrition Collaboration in 2014 to expedite the coordination of future research of nutrition and cancer [140]. Nutritional research is progressively incorporating AI technology to explore the impact of specific dietary components and patterns on the risk of developing disease and the efficacy of treatment [129]. Advances in technology such as AI and machine learning could play a crucial role in personalised nutrition interventions by facilitating the collection, analysis and interpretation of individual dietary and lifestyle assessments, microbiome, genetic and epigenetic data, and other relevant parameters. This would allow for conversion of these insights into practical recommendations. Therefore, further research is required for implementation of these tools into both a clinical and non-clinical setting to improve accessibility to personalised nutrition.

### Misinformation

While individuals’ compliance and enthusiasm are vital, HCPs have an important role in implementing this change safely and effectively. It is essential we utilise the capability of HCPs to recognise and comprehend the importance of diet for cancer prevention and treatment as well as their proficiency in communicating this knowledge. These professionals are trained at conveying complex evidence-based information into understandable and implementable formats for the purpose of optimising patient outcomes. The process of implementing research-driven individualised interventions in a manner which promotes autonomy and self-care is likely to strengthen the relationship between patient and healthcare system while simultaneously providing additional motivation to put this advice into action. The primary advantages of engaging HCPs in this capacity is in their ability to understand the research, assess resource credibility, engage in discussions to aid in making informed individual-focused alterations in guidance, and a knowledge of how these dietary changes may impact other concurrent treatments. As previously highlighted, the breadth and complexity of the information and research available makes attempts to summarise, assess the clinical value, and implement the findings, remarkably challenging. Therefore, there is a significant amount of unsubstantiated and misinterpreted information which is easily accessible [109]. Without trusted professionals to provide this nutritional guidance, individuals may choose to utilise this unverified and potentially unsafe advice.

### Food insecurity

Sufficient access to healthy, faith-appropriate food options presents an additional challenge, particularly for individuals already facing deprivation. Food security in the UK has declined in recent times as a result of job losses and income reductions during the COVID-19 pandemic and the ongoing cost-of-living crisis. Furthermore, a cancer diagnosis can cause significant disruption in work and family life, resulting in the redirection of finances away from healthy food [141]. The resulting food insecurity is associated with increased incidence of cancer and risk of mortality [142–145]. In the UK, 14% of the adult population reported experiencing food insecurity in 2022 [146]. In the United States, studies investigating food insecurity in patients with cancer have demonstrated prevalence rates as high as 55% in certain regions, with a national survey reporting that 28% of cancer patients experience food insecurity [147, 148]. Additionally, research indicates that for the most deprived 20% of UK households to adhere to the EatWell Guide, which outlines the UK Government’s recommendations on healthy eating, they would need to allocate 50% of their disposable income [149, 150]. According to figures from UK charity Turn2us, approximately 4.8 million people in the UK lack at least one essential household appliance, with nearly 2 million people living without a cooker and 2.8 million without a freezer [151]. This highlights that access to and affordability of healthy food constitutes only one aspect of the widespread issue of food insecurity.

While there are many factors contributing to the mounting food insecurity in the UK, there is a growing recognition of the important role the NHS must assume in tackling this health inequity [152]. Primary care is an ideally placed service for addressing this disparity as well as providing dietary advice. Emerging healthcare initiatives are increasingly adopting “food is medicine” interventions for the prevention and management of diet-related disease, resulting in enhanced outcomes, as well as decreased costs and demand on healthcare systems. These interventions incorporate personalised therapeutic diets, which can include tailored meal plans, as well as free or subsidised produce provided through prescriptions [153]. In the UK, these initiatives have also raised awareness of health-oriented dietary choices [154].

**Fig. 4:**
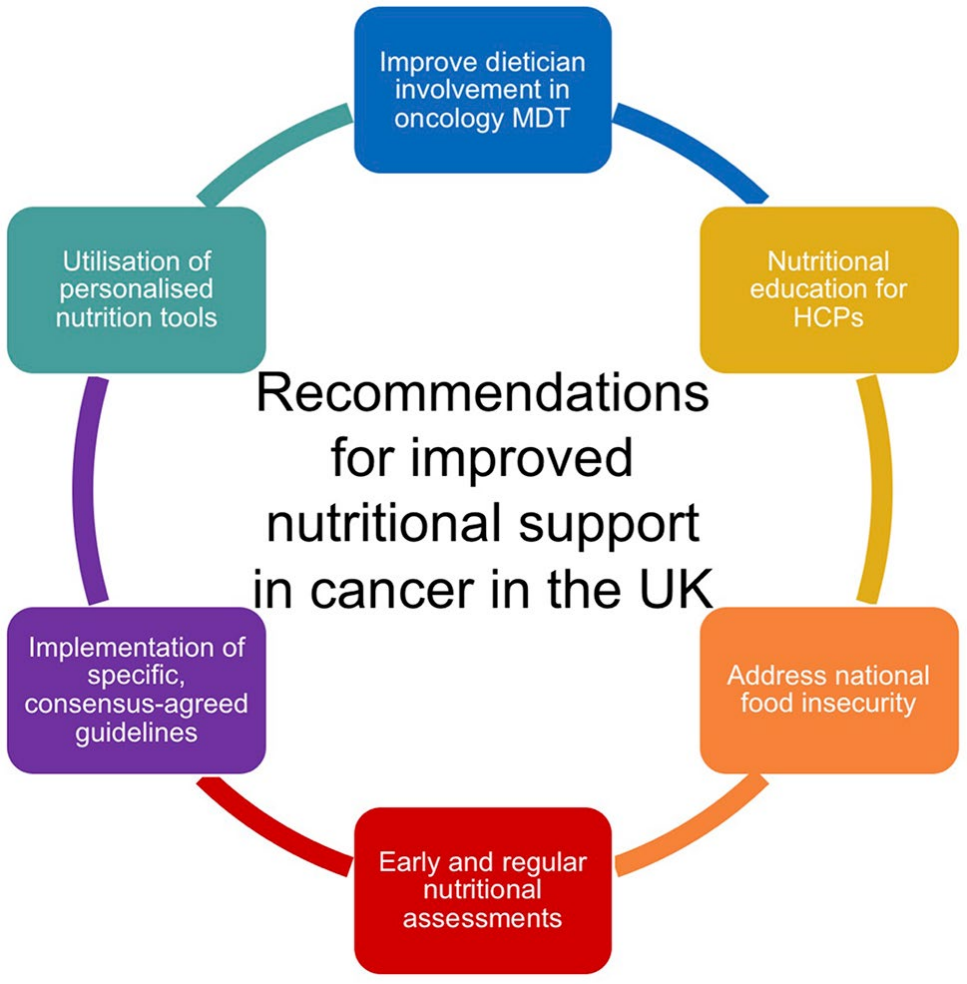
Summary of key recommendations for improved nutritional care for cancer patients and survivors in the UK. The identified areas for improvement involve the refinement of guidelines specificity and adherence to consensus agreed-upon guidance, integration of personalised nutrition tools, involvement of dieticians within oncology MDTs, provision of nutritional education for HCPs and addressing food security issues within the UK

Following the UK’s withdrawal from the European Union (EU), a range of new and amended policies became necessary, impacting various aspects related to nutrition, including agriculture, national food strategies, sustainability, trade and food standards. At the time, the UK obtained approximately one-third of its food supply from the EU. With the National Food Strategy still in its infancy post-Brexit, the COVID-19 pandemic introduced further disruption, impacting food systems worldwide, especially for low-income demographics [155]. Changes in trade and investment agreements can profoundly impact the quality and cost of food, thereby affecting dietary intake and risk of non-communicable diseases such as cancer [156]. Hence, the health consequences stemming from these trade agreements, whether positive or negative, hinge on the political-economic landscape of Britain after Brexit. As predicted, there has been a notable rise in food prices post-Brexit, significantly impacting food security. Research suggests that food prices have increased nearly 25% from 2019 to 2023. Analysis indicates this figure would be closer to 17% without the impact of the UK leaving the EU [157]. Overall, the predominant dietary impacts of Brexit which contribute to an increased risk of cancer include reduced fruit and vegetable consumption and increased red meat intake [158]. Amidst the significant overhaul of trade, agricultural and public policy, there remains an opportunity to improve public health through integration and consideration of public health goals. Policy makers now face the challenge of addressing environmental factors such as climate change, political factors such as Brexit, and socio-cultural factors such as inequalities and other determinants of health, diet and nutrition. However, by prioritising the regulation of structural determinants of health related to nutrition, including policies concerning processed food, alcoholic and sugar-sweetened beverages, and reforming agricultural subsidies to encourage the production and distribution of healthier foods like fruits and vegetables, we can potentially steer the overall impact in a positive direction. See (Fig. 4).

## Conclusions

Cancer remains one of the greatest public health concerns both in the UK and globally. Hence, prioritisation of prevention strategies has become critical, with nutritional changes playing a pivotal role in this approach. Research exploring cancer metabolism and pathogenesis has elucidated the substantial impact of diet in the incidence, progression and prognosis of the disease. The involvement of diet in these processes has been demonstrated across various levels, encompassing genetic, epigenetic and cellular mechanisms. This has prompted exploration into the utilisation of this knowledge for cancer prevention and for the improvement of treatment outcomes. This promising research warrants further investigation with refined, validated and consensus-agreed protocols to better facilitate the incorporation of these findings into clinical practice and public policy. Ensuring greater integration of dietary guidance into public policy and clinical practice is essential to combat the rising incidence and mortality.

Nutrition remains an underutilised and underprioritised factor in the care of cancer patients and survivors, with unsatisfactory numbers of individuals receiving adequate nutritional guidance. There are several barriers identified within the UK which must be addressed. These include insufficient training of HCPs, incorporation of dieticians within the multidisciplinary team, food security, and universally agreed-upon guidelines for prevention, cancer patients and survivors. Both the public and HCPs have displayed a significant desire to rectify these shortcomings. Based on the available research, it appears the most effective method for the integration of dietary changes for these purposes is through a patient-centred, expert-led, personalised nutrition approach. This strategy is likely to yield the most optimal outcomes while preventing engagement with the vast misinformation available.

While this review explores and emphasises the importance of diet in cancer prevention and treatment, it is crucial to recognise this constitutes one piece of the chemoprotective and chemotherapeutic puzzle. The significance of evidence-based clinical treatment protocols and other holistic care strategies, such as physical exercise and smoking cessation, cannot be overstated. The most efficacious method will entail a comprehensive approach integrating optimised medical therapy alongside personalised dietary guidance and other holistic strategies.

## Data Availability

No datasets were generated or analysed during the current study.

## Abbreviations

WCRF: World Cancer Research Fund
ESPEN: European Society for Clinical Nutrition and Metabolism
SNP: Single nucleotide polymorphism
SCFAs: Short chain fatty acids
HCPs: Healthcare professionals
BMI: Body mass index
MDT: Multidisciplinary team
NHS: National Health Service
AI: Artificial intelligence
EU: European Union
